# Cerebral Fructose Metabolism as a Potential Mechanism Driving Alzheimer’s Disease

**DOI:** 10.3389/fnagi.2020.560865

**Published:** 2020-09-11

**Authors:** Richard J. Johnson, Fernando Gomez-Pinilla, Maria Nagel, Takahiko Nakagawa, Bernardo Rodriguez-Iturbe, Laura G. Sanchez-Lozada, Dean R. Tolan, Miguel A. Lanaspa

**Affiliations:** ^1^Division of Renal Diseases and Hypertension, University of Colorado Anschutz Medical Campus, Aurora, CO, United States; ^2^Department of Integrative Biology and Physiology, University of California, Los Angeles, Los Angeles, CA, United States; ^3^Departments of Neurology and Ophthalmology, University of Colorado Anschutz Medical Campus, Aurora, CO, United States; ^4^Department of Nephrology, Rakuwakai Otowa Hospital, Kyoto, Japan; ^5^Department of Cardio-Renal Physiopathology, Instituto Nacional de Cardiología “Ignacio Chávez”, Mexico City, Mexico; ^6^Department of Biology, Boston University, Boston, MA, United States

**Keywords:** fructose, sugar, uric acid, fructokinase, AMP deaminase, mitochondria

## Abstract

The loss of cognitive function in Alzheimer’s disease is pathologically linked with neurofibrillary tangles, amyloid deposition, and loss of neuronal communication. Cerebral insulin resistance and mitochondrial dysfunction have emerged as important contributors to pathogenesis supporting our hypothesis that cerebral fructose metabolism is a key initiating pathway for Alzheimer’s disease. Fructose is unique among nutrients because it activates a survival pathway to protect animals from starvation by lowering energy in cells in association with adenosine monophosphate degradation to uric acid. The fall in energy from fructose metabolism stimulates foraging and food intake while reducing energy and oxygen needs by decreasing mitochondrial function, stimulating glycolysis, and inducing insulin resistance. When fructose metabolism is overactivated systemically, such as from excessive fructose intake, this can lead to obesity and diabetes. Herein, we present evidence that Alzheimer’s disease may be driven by overactivation of cerebral fructose metabolism, in which the source of fructose is largely from endogenous production in the brain. Thus, the reduction in mitochondrial energy production is hampered by neuronal glycolysis that is inadequate, resulting in progressive loss of cerebral energy levels required for neurons to remain functional and viable. In essence, we propose that Alzheimer’s disease is a modern disease driven by changes in dietary lifestyle in which fructose can disrupt cerebral metabolism and neuronal function. Inhibition of intracerebral fructose metabolism could provide a novel way to prevent and treat this disease.

## Introduction

Alzheimer’s disease is the sixth most common cause of death in the United States. The prevalence of dementia is expected to double in the next 20 years, and to affect 81 million people worldwide (Rizzi et al., 2014). Thus, there is a pressing need to identify the cause of Alzheimer’s disease and to establish effective therapies.

Alzheimer’s disease is defined as dementia associated with cerebral atrophy and white matter degeneration with neurofibrillary tangles consisting of hyperphosphorylated tau protein and extraneuronal β-amyloid plaques containing Aβ peptides. A favored hypothesis is that Alzheimer’s disease is mediated by the effects of the amyloid plaques to alter neurological function (the amyloid cascade hypothesis). However, the results of several clinical trials focusing on preventing the amyloid formation and/or degrading amyloid deposits have been disappointing (Anderson et al., 2017; Morris et al., 2018). While the amyloid cascade hypothesis has not been discounted, other hypotheses are now receiving attention, including the role of cerebral insulin resistance and glucose hypometabolism (Neth and Craft, 2017), neuroinflammation (characterized by high cytokine levels at sites of tissue degeneration), synaptic dysfunction, and the role of mitochondrial dysfunction with alterations in intracerebral ATP levels (Demetrius et al., 2014; de la Monte, 2017; Cenini and Voos, 2019). There is also evidence that Alzheimer’s disease may be linked with obesity and diabetes, and with the western diet (Arvanitakis et al., 2004; Gomez-Pinilla and Yang, 2018). However, while obesity and diabetes are risk factors for Alzheimer’s disease (Arnold et al., 2018), Alzheimer’s disease may occur in the absence of these conditions. Therefore, a unifying mechanism for the initiation of Alzheimer’s disease has remained enigmatic.

Herein, we present a unifying hypothesis that brings together prior hypotheses by proposing that intracerebral fructose metabolism may be a key contributing factor for Alzheimer’s disease. We will present evidence of how this hypothesis can account for known risk factors and how it can explain Alzheimer’s disease pathophysiology. To understand this hypothesis, we will first review recent breakthroughs in our understanding of fructose metabolism and then relate this to the development of Alzheimer’s disease.

## Recent Insights in Fructose Metabolism

Two major simple sugars present in our diet are glucose and fructose. Polymers of glucose make up starch and is a primary component of high glycemic carbohydrates, such as bread, rice, and potatoes. Fructose, or fruit sugar, is the primary sugar present in fruits and honey. Two common added sweeteners in our diet are sucrose (table sugar) and high fructose corn syrup (HFCS). Both of these sweeteners contain glucose and fructose, with the glucose and fructose bonded as a disaccharide in sucrose, and with both mixed as monosaccharides in different ratios (but usually 55:45 fructose: glucose) in HFCS. Together, intake of sugar and HFCS comprises 15% of the total calories ingested in a western diet but may reach 25% of the total calories in different populations (Yang et al., 2014). These sweeteners are also present in 70% of processed foods in supermarkets (Ng et al., 2012).

### Biological Effects of Fructose Are Distinct From Glucose

**Figure 1:**
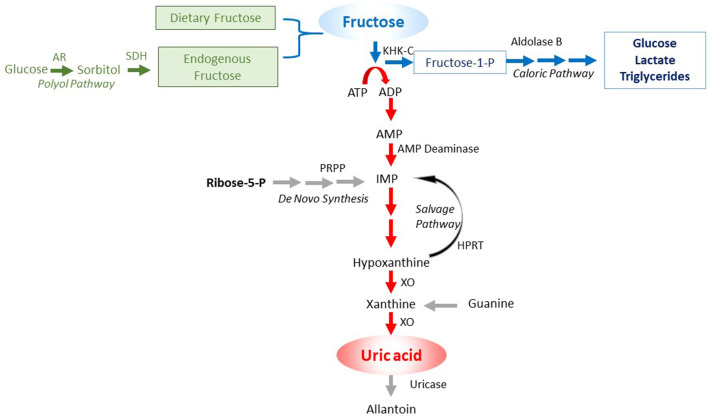
Purine degradation pathway induced by fructose. Fructose can come from the diet or be endogenously produced by the polyol pathway. In turn, the metabolism of fructose by fructokinase C (KHK-C) can lead to ATP consumption, with intracellular phosphate depletion, resulting in activation of AMP deaminase (AMPD) that eventually leads to the production of uric acid. Key: green color shows the sources of fructose, the blue color the caloric pathway of fructose metabolism, and the red color the pathway involving nucleotide degradation that activates the survival pathway.

An important insight has been the discovery of unique differences in glucose and fructose metabolism that translates into differences in biologic function. While glucose acts as an energy-producing fuel, like most nutrients, fructose appears to be used as an energy-storing fuel (Johnson et al., 2020). This feature of fructose is due to its unique metabolism that decreases energy (adenosine triphosphate; ATP) within the cell (Maenpaa et al., 1968; van den Berghe et al., 1977). The mechanism is mediated by a specific enzyme, fructokinase C (also known as ketohexokinase C, or KHK-C) that phosphorylates fructose to fructose-1-phosphate so rapidly that intracellular phosphate and ATP levels fall. In turn, the low intracellular phosphate activates adenosine monophosphate (AMP) deaminase, resulting in the stepwise degradation of AMP to inosine monophosphate (IMP) and eventually uric acid (Figure 1). Activation of AMP deaminase-2 (AMPD2) results in a removal of AMP, thereby reducing the ability of the cell to replenish ATP levels, while stimulating the production of uric acid that inhibits AMP-activated protein kinase (AMPK), thereby reducing ATP generation (Lanaspa et al., 2012a; Cicerchi et al., 2014). The ability of fructose to reduce intrahepatic ATP levels and increase intracellular and serum uric acid levels occurs with the ingestion of soft drinks (Le et al., 2012; Bawden et al., 2016). In contrast, other major food groups (glucose, protein, and fats) act to increase energy levels in the cell.

The fall in intracellular ATP levels with the generation of uric acid activates various alarm signals, including mitogen-activated protein kinases (MAPK) and nicotinamide adenine dinucleotide phosphate (NADPH) oxidase (Maenpaa et al., 1968; Kang et al., 2002; Lanaspa et al., 2012b; Sanchez-Lozada et al., 2012). A burst of intracellular oxidative stress occurs, especially in mitochondria (Lanaspa et al., 2012a). Fructose magnifies this effect by blocking the Nrf2-Keap1 antioxidant pathway (García-Arroyo et al., 2019a). A reduction in mitochondrial function occurs, with a blockade in fatty acid beta-oxidation (due to inhibition of enoyl CoA hydratase) and a shift to glycolysis (Lanaspa et al., 2012b; Softic et al., 2019). The mitochondrial enzyme, aconitase-2, is inhibited, leading to citrate accumulation that passes into the cytoplasm where it stimulates lipogenesis. Lactate generation occurs that enhances mitochondrial dysfunction (San-Millán and Brooks, 2017). As a consequence, total energy production stays low, with much of the energy being produced by aerobic glycolysis (Warburg effect; Nakagawa et al., 2020). This helps cells maintain the low energy state in concert with the fall in intracellular ATP levels.

Many biologic effects associated with energy depletion are mediated, in part, by the generation of intracellular uric acid. Uric acid, while an anti-oxidant in the extracellular environment, is pro-inflammatory in the intracellular environment, and activates p38 MAP kinase and NF-κB, induces NADPH oxidase-induced oxidative stress, and stimulates the production of chemotactic factors, vasoconstrictive substances (renin-angiotensin system), and growth factors (Johnson et al., 2013). Uric acid also reduces endothelial nitric oxide bioavailability *via* several mechanisms. Concerning fructose biology, the mitochondrial oxidative stress, the induction of insulin resistance, and the inhibition of AMPK are all partially dependent on the uric acid (Nakagawa et al., 2006; Lanaspa et al., 2012b; Cicerchi et al., 2014).

The ability of fructose metabolism to lower energy in the cell requires the presence of both fructokinase C (KHK-C) and fructose. Fructose may come from the diet, especially from sugar and HFCS-containing foods. However, fructose is also generated in tissues by activation of the aldose reductase (AR)-sorbitol dehydrogenase (SDH) pathway (polyol pathway), which converts glucose to sorbitol (*via* AR) and then sorbitol to fructose by SDH (Table 1). The rate-limiting enzyme is AR, but it can be induced by a variety of mechanisms, including ischemia, hyperosmolarity, alcohol, hyperglycemia, and fructose and uric acid themselves (Johnson et al., 2020). AR is also induced and AR activity is increased in the brain and other organs with aging (Cao Danh et al., 1984; Kwee et al., 1991). Certain foods that do not contain fructose also activate AR and stimulate endogenous fructose production, including high glycemic carbohydrates, salty foods, and alcohol (Lanaspa et al., 2013, 2018; Wang et al., 2020).

**Table 1:** Sources of dietary and endogenous fructose.

**Dietary Fructose**
Honey and Fruits
Added Sweeteners (Sucrose, HFCS)
**Endogenous Fructose (Requires induction of Aldose Reductase)**
Foods
High glycemic carbohydrates (glucose, starch, potatoes, rice, bread)
Salty Foods
Ischemia or Hypoxia
Tissue Injury (Trauma, Vascular Injury)
Hyperosmolarity
Dehydration
Hyperuricemia
Aging

The enzyme, KHK-C, is also regulated. Under normal conditions, KHK-C is primarily located in the small bowel intestinal epithelium, the hepatocyte, and the kidney proximal tubular cells, but it is also present in the pancreatic islets, the adipocytes, the vascular endothelium, and the brain (including the hippocampus and hypothalamus; Diggle et al., 2009; Oppelt et al., 2017; Song et al., 2017). KHK-C expression is enhanced by fructose or high uric acid levels (Roncal-Jimenez et al., 2011; Lanaspa et al., 2012c). KHK-C can also be induced in ischemic tissues, such as the heart (Mirtschink et al., 2015).

In summary, fructose does not simply come from the diet but is also produced in the body, and the ability to metabolize fructose is regulated.

### Fructose Metabolism Activates an Evolutionary-Based Survival Pathway

Animals that are starving activate behavioral and metabolic changes to aid survival once fat stores are depleted. This includes foraging behavior, a reduction in energy production, and the development of insulin resistance (which reduces glucose uptake in muscle, thereby favoring preferential uptake by the brain) (Koffler and Kisch, 1996; Cahill, 2006). To avert starvation, animals store fat in preparation for times of food shortage, such as before hibernation, long-distance migration, or nesting. One approach used by many animals is to ingest fructose-rich foods, such as from fruits and honey, as the fall in energy within the cell triggers the same behavioral and metabolic effect as starvation and thereby acts as an alarm signal for the host to store fat (Johnson et al., 2013, 2020).

**Figure 2:**
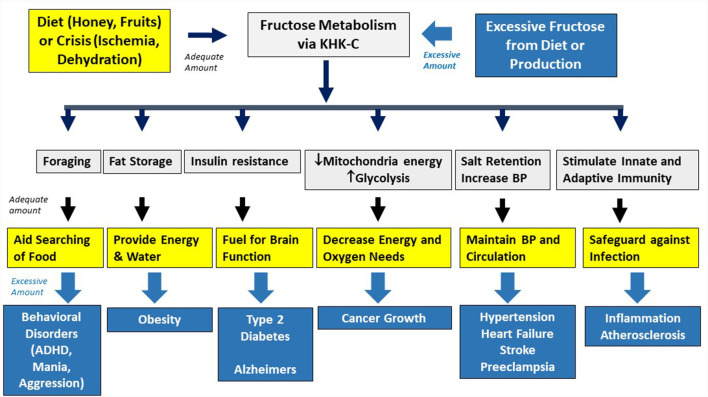
Physiological and pathological effects of fructose. Fructose is used in nature as an innate survival pathway that helps protect animals from food and water shortage. In contrast, excessive intake of fructose-containing sugars overactivated this pathway, resulting in various metabolic disorders.

Fructose metabolism stimulates several survival pathways (Figure 2).

#### Foraging and Bingeing Behavior

While mice generally prefer glucose over fructose when it is provided chronically, when sugar is given intermittently, fructose causes a stronger bingeing response (Rorabaugh et al., 2015). This is associated with hunger responses mediated by the release of orexin from the lateral hypothalamus and is distinct from that observed with glucose (Rorabaugh et al., 2014). The mechanism could involve the depletion of ATP in the hypothalamus (Lane and Cha, 2009). Over time, animals develop leptin resistance that drives excessive food intake while reducing the oxidation of fat (Shapiro et al., 2008). Fructose also stimulates the production of vasopressin (Song et al., 2017) that results in urinary water retention; however, the retained water passes from the extracellular space into the cell and likely binds newly-made glycogen, resulting in continued thirst (Johnson et al., 2020). Degradation of ATP to uric acid may also play a role in increasing locomotor activity (Barrera et al., 1989), exploratory behavior, and impulsivity (Sutin et al., 2014), all features needed when foraging for food and water (Robin et al., 1998).

Studies in humans have largely confirmed these findings. Fructose administration has unique effects on attention and reward centers resulting in more hunger and desire for sweet foods than glucose (Luo et al., 2015) and which is linked with reduced cortical activity involved in the control of behavior by blood oxygen level-dependent (BOLD) MRI (Purnell et al., 2011).

#### Increasing Fat Stores

In addition to stimulating hunger, thirst, and foraging, fructose preferentially increases the storage of fat, including in the liver, blood (triglycerides), and adipose tissues, thereby providing stored energy as well as metabolic water in times of need (Johnson et al., 2016). The primary mechanism appears to be the induction of mitochondrial oxidative stress that leads to lipogenesis (from blocking aconitase) and reduced beta fatty acid oxidation (from blocking enoyl CoA hydratase; Lanaspa et al., 2012a,b; Softic et al., 2019).

#### Protecting Against Hypoxia

The fructose-induced switch from mitochondrial function to glycolysis protects by minimizing oxygen demands and reducing energy needs. Endogenously produced fructose is key for the survival of the naked mole-rat in its hypoxic burrows (Park et al., 2017) and is also produced by the placenta of many species (including humans) where it may protect the developing fetus before the establishment of blood supply (Jauniaux et al., 2005).

#### Maintenance of Blood Pressure and Activation of Inflammatory Pathways

Fructose also has effects on blood pressure, likely mediated in part by activation of vasopressin, the renin-angiotensin system, and other vasoconstrictor pathways. Fructose also stimulates sodium reabsorption in the proximal tubule (Cabral et al., 2014). Uric acid, generated by the fructose, increases blood pressure responses by reducing endothelial nitric oxide, stimulating oxidative stress, and activating the renin-angiotensin system (Sanchez-Lozada et al., 2020). There is also a very active stimulation of innate immunity likely mediated by the uric acid (Joosten et al., 2020). These are all protective systems to aid survival in extreme conditions.

#### Brain Protection

Insulin resistance also develops, which reduces uptake of glucose by the skeletal muscle, thereby leading to its preferential uptake by the brain.

Fructose, either from the diet or endogenously produced, is used by many species to aid survival (Johnson et al., 2020). Of interest, mutations that could enhance this pathway have occurred in various species, including humans (Johnson and Andrews, 2015). One mutation involved the uricase gene. Uricase is an enzyme that breaks down uric acid to allantoin and is the primary way most mammals regulate their uric acid levels. Our early ancestors also had uricase, but as they lived primarily on fruit, they were still able to eat enough fructose to provide the key energy they needed. Unfortunately, during the Miocene Epoch, there was global cooling that affected fruit availability, especially during the cooler months, and as the fruit became scarce, many apes starved to extinction. During this time a mutation in uricase occurred which amplified the uric acid response during fructose metabolism. This provided a survival advantage by resulting in enhanced storage of fat in response to fructose. This mutation, which is present in all humans, amplifies our metabolic responses to fructose compared to most other mammals (Johnson and Andrews, 2015).

### Fructose and the Metabolic Syndrome

In the setting of most hunter-gatherer diets, the intake of fructose is limited to fruits and honey and obesity, and metabolic syndrome is rare. Serum uric acid levels also tend to be low and blood pressure is in the normal range (Johnson et al., 2005). Under these conditions, the amount of fructose ingested is relatively low, and the survival pathway is utilized to protect against starvation rather than to cause obesity. It is noteworthy that fruits and honey contain other nutrients such as flavonoids that have the neuroprotective capacity (Gomez-Pinilla and Nguyen, 2012).

However, the remarkable increase in sugar and HFCS intake has led to excessive and chronic activation of this pathway, resulting in increased risk for metabolic syndrome, obesity, and diabetes (Figure 2; Johnson et al., 2013). The risk is particularly high with liquid sugars, such as soft drinks, as the high fructose content coupled with rapid intake leads to high concentrations in the liver that can lead to more profound intracellular energy depletion (Sundborn et al., 2019). While dietary fructose is a major source of fructose, endogenous production of fructose may also drive the metabolic syndrome, such as in response to high glycemic carbohydrates and high salt diets (Lanaspa et al., 2013, 2018). There is also evidence that excessive fructose increases the risk for hypertension (Jalal et al., 2010), systemic inflammation (Cox et al., 2011), and cancer metastases and growth (Goncalves et al., 2019; Nakagawa et al., 2020).

Over time, chronic mitochondrial oxidative stress causes impaired mitophagy with the accumulation of damaged mitochondria and fewer functional mitochondria (Shefa et al., 2019), thereby affecting overall energy production and metabolism and causing increased reliance on glycolysis. While low fructose diets or low salt diets can help recover mitochondrial numbers (Hernandez-Rios et al., 2013), if energy levels cannot be maintained, such as by impaired glycolytic compensation, then cell death may result. Chronic mitochondrial oxidative stress may also have a role in the aging process and aging-associated disease (Sun et al., 2016), and, interestingly, low-grade endogenous fructose metabolism has also been linked with certain aging-related pathologies (Roncal-Jimenez et al., 2016).

Next, we will discuss how chronic activation of this pathway may underlie the pathogenesis of Alzheimer’s disease.

## Fructose Metabolism and Alzheimer’S Disease

**Figure 3:**
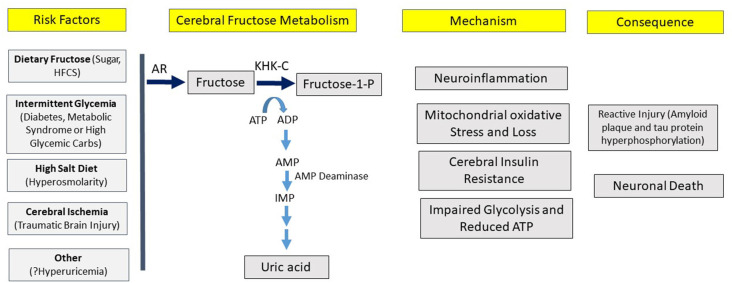
Hypothesis for the development of Alzheimer’s disease. According to the hypothesis, various risk factors can induce cerebral fructose metabolism that can initiate a variety of processes, leading to the pathological and clinical manifestations of Alzheimer’s disease.

Here we present the hypothesis that overactivation of the fructose “survival” pathway in the brain may be a driving cause of Alzheimer’s disease, similar to the data that supports overactivation of the fructose pathway systemically plays a role in the metabolic syndrome. A key aspect of the hypothesis is that one may be able to have overactivation of the fructose system in the brain despite minimal activation systemically, and vice versa. The strength of the hypothesis is based on a series of observations that link various aspects of the epidemiology, clinical presentation, and biology into one pathway (Table 2; Figure 3).

**Table 2:** Evidence for cerebral fructose metabolism playing a role in Alzheimer’s disease.

• Dietary Intake of Fructose is Associated with Cognitive Dysfunction in Animals and Humans
• Many Risk Factors for Alzheimer’s Disease Activate Endogenous Fructose Metabolism
• Fructose Metabolism is Active in the Brain of Alzheimer’s Disease Patients
• Cerebral Fructose Metabolism may have a Role in the Alzheimer Disease Pathogenesis
Cerebral Insulin Resistance and Cerebral Glucose Hypometabolism
Mitochondrial Dysfunction and Energy Depletion
Neuroinflammation
Amyloid Production and Tau Protein Hyperphosphorylation

### Dietary Intake of Fructose Is Associated With Cognitive Dysfunction in Animals and Humans

There has been substantial interest in the role of diet, especially fructose-containing sugars, and it’s potential for affecting cognition and the risk for Alzheimer’s disease (Beilharz et al., 2015).

#### Animal Models

Intake of sugar or HFCS has been reported to induce cognitive dysfunction in laboratory rats and mice. For example, the administration of sucrose or HFCS causes inflammation in the hippocampus in association with memory deficit in rats, and this defect is worse in rats fed HFCS that has higher fructose content (Hsu et al., 2015). Sugar provided in liquids (liquid sugar) tends to induce more cognitive dysfunction than when sugar is administered in solid form, or of diets high in fat, and occurs independently of weight gain (Beilharz et al., 2016). Chronic feeding of sugar to adolescent rats also increases the risk for the development of cognitive dysfunction in adulthood (Reichelt et al., 2015).

Chronic fructose consumption in rodents disrupts hippocampal pathways associated with cell energy metabolism involving peroxisome proliferation-activated receptor gamma coactivator 1-alpha (PGC-1α), mitochondrial transcription factor A (TFAM) and sirtuin 1, and synaptic plasticity modulators such as cAMP response element-binding protein (CREB; Agrawal et al., 2016).

Cognitive dysfunction in fructose-fed rats is associated with substantial epigenomic and transcriptional reprogramming in both the hypothalamus and hippocampus including alterations in the *Bgn* and *Fmod* extracellular matrix genes (Meng et al., 2016). These studies suggest that fructose may disrupt the interface between cell metabolism and synaptic plasticity, making the brain susceptible to neurological disorders.

The effect on memory-driven by the fructose component of sugar is amplified by diets low in omega-3 fatty acids (Agrawal and Gomez-Pinilla, 2012). Combining fructose with saturated fats (coconut oil) causes worse memory defects than when combined with fats rich in omega-6 polyunsaturated fats (such as soybean oil; Lin et al., 2017). Furthermore, supplementation of omega-3 fatty acids can improve both fructose-induced metabolic syndrome and fructose-induced cognitive defects (Meng et al., 2016). In contrast, high-fat diets alone usually do not cause memory deficits (such as spatial or short term memory testing) unless combined with fructose (Cordner and Tamashiro, 2015).

#### Human Studies

Sugar intake, especially soft drinks, is also associated with cognitive dysfunction. In the Framingham Heart Study, intake of soft drinks and fruit juices was associated with dose-dependent reductions in the total brain and hippocampal volume along with worse episodic memory (Pase et al., 2017). Total sugar intake was associated with worse cognition in older community dwellers in Malaysia, while the intake of natural fruits appeared protective (Chong et al., 2019). Similar findings were reported with a population from Puerto Rico and in this study intake of liquid sugars was especially linked with cognitive dysfunction while natural sources of fructose (such as fruits) were not (Ye et al., 2011). Another study reported that cognitive dysfunction in elderly subjects is increased by high carbohydrate diets compared to diets high in fat or protein (Roberts et al., 2012). Likewise, Mediterranean style diets low in sugar and high in omega-3 fatty acids appear to be protective (Berti et al., 2015). Recently, a clinical study placed subjects on a high-sugar, high-fat diet with a more healthy diet, and found that at 4 days an impairment in short term memory and hippocampal function could be shown in the high sugar, high-fat diet group (Attuquayefio et al., 2017). The observation that total and liquid sugars are more likely associated with cognitive dysfunction is consistent with studies showing that liquid sugars cause more significant ATP depletion and metabolic effects, while the negative studies with natural fruits are also consistent given the presence of antioxidants and flavonols in fruits that are known to counter fructose effects (Sundborn et al., 2019).

These associations may begin early in life. Maternal intake of soft drinks during pregnancy as well as soft drink intake in early childhood is associated with cognitive dysfunction in children (Cohen et al., 2018). Children with a higher intake of refined carbohydrates also show lower nonverbal intelligence scores (Abargouei et al., 2012). Our group also reviewed the strong experimental, pathophysiological, and clinical association of chronic sugar intake with attention deficit hyperactivity syndrome (Johnson et al., 2011).

### Many Risk Factors for Alzheimer’s Disease Activate Endogenous Fructose Metabolism

As mentioned, humans can produce fructose (fructoneogenesis), and the only known way is *via* activation of aldose reductase (AR) of the polyol pathway (Figure 1). If the fructose pathway is key for the development of Alzheimer’s disease, one might expect some evidence of a relationship between conditions associated with endogenous fructose production (Table 1) with the development of dementia.

#### Obesity, Metabolic Syndrome, and Diabetes

Metabolic syndrome, hypertriglyceridemia, type 2 diabetes, and obesity are all risk factors for Alzheimer’s disease (Seaquist, 2010; Solfrizzi et al., 2011; —, 2005; Rosales-Corral et al., 2015; Anjum et al., 2018). All of these conditions have been linked with increased dietary intake of sugar and HFCS (Johnson et al., 2013; Malik and Hu, 2015), however, experimental evidence also links these conditions with endogenous fructose production (Lanaspa et al., 2013, 2018). The high glycemic state associated with diabetes also activates AR in multiple tissues associated with endogenous fructose production, including the brain (Stewart et al., 1967). Poor glycemic control in subjects with type 2 diabetes has also been found to acutely worsen memory (Greenwood et al., 2003). The amount of endogenous fructose produced in diabetes can be significant, for blocking AR prevents nonalcoholic fatty liver disease (NAFLD) in diabetic mice (Qiu et al., 2012).

#### High Glycemic Diets

Clinical studies have linked intake of refined carbohydrates (which includes both sugar and high glycemic carbohydrates such as bread and rice) with dementia (Abargouei et al., 2012; Roberts et al., 2012). While one might interpret this to mean it is due to the inclusion of fructose-containing foods, there is an experimental study showing that maltodextrin can cause memory deficits in rats (Kendig et al., 2014). Maltodextrin does not contain fructose, but rather is similar to starch and is broken down to glucose. However, our group has reported that intake of glucose, a high glycemic carbohydrate, results in activation of AR in the liver that results in endogenous fructose production that then drives the development of the metabolic syndrome and insulin resistance (Lanaspa et al., 2013). Studies in humans have also shown that the ingestion of glucose causes a rise in serum glucose that is then converted to fructose in the brain (Hwang et al., 2017). These studies suggest high glycemic carbohydrates may also be a potential risk factor for Alzheimer’s disease.

#### High Salt Diets

While the western diet is associated with high sugar content, there has also been a remarkable increase in salt intake over the last century. Today the average intake of salt is approximately 8–10 grams/day. For a long time, diets high in salt were thought to be primarily of interest for their potential role in causing high blood pressure and cardiovascular disease (He and MacGregor, 2011). More recently high salt intake has been associated with obesity, metabolic syndrome, and diabetes (Libuda et al., 2012; Lanaspa et al., 2018). Experimental studies suggest that these effects may be mediated by the effect of dietary salt to induce hyperosmolarity (Suckling et al., 2012; Kanbay et al., 2018), which is known to stimulate AR and endogenous fructose production (Ko et al., 1997). Our group reported that a high salt diet-induced endogenous fructose production in multiple tissues in mice, including the liver and hypothalamus, with the development of metabolic syndrome (Lanaspa et al., 2018). If fructose metabolism was inhibited, neither metabolic syndrome nor elevated blood pressure develops (Lanaspa et al., 2018).

High salt diets are now being linked with dementia. High salt diets initially stimulate exploratory behavior in mice, but later it leads to a reduction in the short term and long-term memory with oxidative stress to the hypothalamus (Liu et al., 2014; Ge et al., 2017; Guo et al., 2017) that tends to be worse in older animals (Chugh et al., 2013). Moreover, a high salt diet induces hyperphosphorylation of tau protein in the hypothalamus (Faraco et al., 2019). High salt concentrations also increase amyloid Aβ peptides in cultured embryonic kidney cells (Cheng et al., 2015).

#### Trauma

Traumatic brain injury increases the risk of developing Alzheimer’s disease (Al-Dahhak et al., 2018). Based on experimental studies, this involves disruption of the microvasculature, leading to local ischemia that triggers oxidative stress (Leker and Shohami, 2002). One mechanism by which ischemia causes oxidative stress is by stimulating AR and fructose generation (Andres-Hernando et al., 2017). Traumatic brain injury activates pathways involved in lipid metabolism in the hypothalamus and liver (Rege et al., 2019). Dietary fructose intake also magnifies the oxidative stress associated with traumatic brain injury, resulting in worse spatial memory deficits, depression in mitochondrial function, and development of cerebral insulin resistance (Agrawal et al., 2016).

#### Alcohol

Alcohol, while capable of inducing dementia on its own, also predisposes subjects to Alzheimer’s disease (Venkataraman et al., 2017). Alcohol intake, by increasing serum osmolality, can activate AR and endogenous fructose production in the liver in both humans and experimental animal models (Wang et al., 2020). Blocking AR has also been reported to inhibit the development of hepatic steatosis in response to alcohol in animal models (Shi et al., 2017). Whether blocking AR is beneficial in alcohol-induced models of dementia has not been tested, to our knowledge.

#### Aging

Aging is well known to be associated with increased risk for cognitive decline and the development of Alzheimer’s disease. Of interest, AR expression and activity are increased in the aging brain and this is associated with low intracellular phosphate (which activates AMPD2) and increased sorbitol levels (Cao Danh et al., 1984; Kwee et al., 1991). Our group also found evidence that low-grade endogenous fructose production may be causing aging-associated pathology in the kidney. Specifically, aging-associated kidney disease did not occur in mice lacking fructokinase who were being fed with a normal chow that had minimal (<5%) fructose (Roncal-Jimenez et al., 2016).

#### Genetics

Alzheimer’s disease is associated with the apolipoprotein E4 (Apo E4) genetic polymorphism, as well as numerous other genes identified through genome-wide associated screening (GWAS; Beecham et al., 2014). Humans administered HFCS show elevations of apolipoprotein E postprandially in addition to developing hypertriglyceridemia (Price et al., 2018). More importantly, a nutrigenomics study found that fructose consumption in rodents promoted selective transcriptomic and epigenomic reprogramming in brain regions related to cognitive processing such as the hippocampus. Fructose signature genes found in rats overlapped with top genes associated with lipid biology and energy metabolism that have been identified as risk factors for Alzheimer’s disease by GWAS studies (Meng et al., 2016).

#### Dietary Fructose

An interesting question is how the intake of sugar and HFCS can induce changes in cognition when most of the fructose is removed by the intestines and liver before reaching the systemic circulation (Jang et al., 2018). Typically serum levels of fructose are quite low compared to glucose, and following the intake of a soft drink is approximately 5 mg/dl (Le et al., 2012). Indeed, studies utilizing labeled fructose suggest only 1–2% reaches the brain (Oldendorf, 1971).

While it is possible that such low concentrations of dietary fructose could be having a direct role in brain function, it is also possible that the metabolism of fructose systemically might lead to the release of factors or neural stimuli that could affect brain metabolism and function. One possibility is that it may be inducing endogenous fructose production and metabolism in the brain. For example, sugar intake can upregulate aldose reductase (García-Arroyo et al., 2017), SDH (García-Arroyo et al., 2017), fructose transporters (Glut5; Roncal-Jimenez et al., 2011), and fructokinase (KHK-C; Korieh and Crouzoulon, 1991; Roncal-Jimenez et al., 2011; Lanaspa et al., 2012c) and xanthine oxidase (García-Arroyo et al., 2019b) through a positive feedback system. That this may occur in the brain is suggested by a study reporting that dietary fructose can increase expression of the fructose transporter, Glut5, in the brain (including the hypothalamus) of rats (Shu et al., 2006). One potential mechanism could be by the release of uric acid into the circulation following fructose metabolism in the liver (Perheentupa and Raivio, 1967), as uric acid can pass freely across the blood-brain barrier (Shao et al., 2016) where it could stimulate expression of AR and KHK-C (Lanaspa et al., 2012c; Sanchez-Lozada et al., 2019). Another possibility is that the fall in ATP in the liver can mediate CNS effects, possibly by the release of factors such as FGF21 or by effects on the vagal nervous system (Friedman, 2007; Talukdar et al., 2016).

### Fructose Metabolism Is Active in the Brain of Alzheimer’s Disease Patients

Figure 1 reviews the steps involved in fructose generation and metabolism, focusing on the KHK-C dependent purine degradation pathway that leads to intracellular ATP depletion.

#### Endogenous Fructose Produced in Alzheimer’s Disease Brains

Aldose reductase is expressed in neurons, including in the hippocampus (Picklo et al., 2001; Hwang et al., 2017). Activation of AR with the generation of fructose has been shown in the brain following dehydration in rats (Song et al., 2017) as well as following glucose loading in humans (Hwang et al., 2017). Importantly, there is evidence for endogenous fructose production in Alzheimer’s disease patients, with intracerebral levels of sorbitol and fructose 3–5-fold greater than normal (Xu et al., 2016). While the expression of AR does not change in subjects with Alzheimer’s disease, the fact that there is a decrease in neuron numbers suggests a relative increase in AR expression and activity per neuron (Picklo et al., 2001).

#### Purine Degradation Pathway Is Activated in Alzheimer’s Disease Brains

Once fructose is generated, it can be metabolized by KHK-C, triggering a fall in intracellular phosphate and ATP that triggers the purine degradation pathway, generating AMP that is metabolized by AMPD2 to produce IMP and ammonia (van den Berghe et al., 1977; Figure 1). Fructose can also be metabolized by hexokinase, and this may be a significant route of metabolism in the normal rat brain cortex (Hassel et al., 2015). However, it is preferentially metabolized by KHK-C if this enzyme is present. Indeed, KHK-C is expressed in the brain, including the hypothalamus and hippocampus (Oppelt et al., 2017). To date, no studies have examined the expression of KHK-C in Alzheimer’s patients. However, the first enzyme in the purine degradation pathway in fructose metabolism is AMPD2, and studies have reported both increased expression and activity of AMPD-2 in Alzheimer’s patients (Sims et al., 1998). One of the products of AMPD2 is ammonia, and several studies have reported increased blood ammonia levels in Alzheimer’s patients (Adlimoghaddam et al., 2016; Jin et al., 2018). One study also reported that ammonia production is increased in the brain of Alzheimer’s patients (Hoyer et al., 1990).

The IMP generated by AMPD2 also continues to degrade, eventually generating hypoxanthine that can either be recycled back to IMP or can be further degraded by xanthine oxidase to generate intracellular uric acid. As a consequence, one might expect to observe higher uric acid levels in the brains of Alzheimer’s patients, but the only study to date showed no difference from age-matched controls (McFarland et al., 2013). However, once energy stores are depleted (as occurs in Alzheimer’s subjects), there may not be enough substrate for further uric acid formation.

### A Potential Mechanism for Alzheimer’s Disease

We hypothesize that Alzheimer’s disease is a relatively modern disease of western culture and that it represents a disorder of chronic fructose metabolism (Figure 3).

#### Step 1: Endogenous Fructose Is Produced in the Brain

We suggest that disease begins when we unconsciously activate the fructose survival pathway by eating excess sugar and HFCS, with the greatest risk being from drinking soft drinks in which large amounts of fructose are ingested rapidly, resulting in more severe ATP depletion. Recurrent intake of sugar slowly increases expression and activity of enzymes involved in both endogenous fructose production and metabolisms, such as AR, SDH, KHK-C, and xanthine oxidase in various tissues, including the brain. Other foods may also activate endogenous production of fructose, including salty foods, high glycemic carbohydrates, and alcohol. It is also possible to partially bypass the fructose pathway by eating umami-rich foods, as the glutamate can be metabolized to uric acid (Feigelson and Feigelson, 1966) *via* an AMPD-dependent pathway while the purines such as IMP can be degraded to uric acid in the gut and liver. Other mechanisms may also increase intracerebral production of fructose, such as episodes of postprandial hyperglycemia in the subject with metabolic syndrome, persistent hyperglycemia in type 1 or type 2 diabetic subjects, or ischemia following traumatic brain injury. Subjects with marked hyperuricemia may also carry some level of risk, although local uric acid production may be more important.

#### Step 2: Fructose Is Metabolized, Setting off an Innate Survival Pathway

As the brain starts metabolizing fructose by KHK-C, there is a transient fall of ATP in the neurons with activation of AMPD2 generating ammonia and IMP. As mentioned, AMPD2 activity and expression are high in the brains of Alzheimer’s subjects (Sims et al., 1998) as are blood and CNS ammonia levels (Hoyer et al., 1988, 1990). While the ammonia may contribute to some cognitive dysfunction, the IMP is then broken down to uric acid, which may be more important to the pathology, given that intracellular uric acid stimulates mitochondrial oxidative stress and inflammation.

#### Step 3: Intracellular Uric Acid Induces Neuroinflammation

An important article by Shao et al. (2016) demonstrated the importance of uric acid as a neuroinflammatory substance. The authors first showed that the induction of hyperuricemia in rats (using a uricase inhibitor) could induce cognitive defects in addition to its known effects on impulsivity (Sutin et al., 2014) and this was associated with hippocampal inflammation with NFκB and Toll-like receptor 4 activation and inflammatory cytokine production (interleukin 1b and interleukin 6; Shao et al., 2016). The hypothalamic inflammation could be reproduced by direct injection of uric acid into the hippocampus whereas allantoin (the product of uricase) did not. Uric acid also caused cellular activation of primary hippocampal cells and culture. Finally, humans with hyperuricemia showed gliosis of their hippocampal region by MRI (Shao et al., 2016). Another study reported that uric acid could potentiate Aβ amyloid peptides in inducing neuronal death in cell culture (Desideri et al., 2017). These results are consistent with a study that used functional MRI and demonstrated that hyperuricemic individuals (especially males) showed poorer cognition and less spontaneous electrical activity in the basal ganglia, including the putamen and pallidum (Lin et al., 2019).

The argument that uric acid may have a role in Alzheimer’s disease may seem inconsistent with reports that Alzheimer’s disease is associated with lower serum uric acid levels (Euser et al., 2009; Ye et al., 2016). However, other studies have reported that hyperuricemia may predict dementia (Khan et al., 2016). Indeed, hyperuricemia has been associated with deficits in memory and word processing in older adults (Schretlen et al., 2007), and with white matter, hyper-intense signaling suggestive of ischemic pathology (Schretlen et al., 2007). Hyperuricemia was also associated with cognitive dysfunction in subjects with chronic kidney disease (Afsar et al., 2011) and worse cognition and white matter disease in older adults in the Rotterdam study (Verhaaren et al., 2013).

We believe the reason lower serum uric acid levels are common in Alzheimer’s patients is because serum uric acid also reflects overall nutrition status (Beberashvili et al., 2016), and it is known that subjects with Alzheimer’s disease often lose a significant amount of weight even before they manifest with dementia (Stewart et al., 2005; Johnson et al., 2006). Another argument in support of the importance of uric acid in the disease process comes from two epidemiological studies investigating whether lowering serum and intracellular uric acid levels with allopurinol protects against the development of dementia. A longitudinal Taiwanese study found that subjects with gout who were being treated with urate-lowering therapy had a 30% lower risk for developing dementia than did untreated subjects with gout or controls (Hong et al., 2015). In another study using Medicare claims data, subjects taking allopurinol or febuxostat showed a reduced risk for dementia with higher doses of these uric acid lowering drugs (Singh and Cleveland, 2018).

#### Step 4: Cerebral Insulin Resistance and Glucose Hypometabolism

The fructose survival pathway was meant to reduce energy needs and encourage food intake, and the development of insulin resistance was a protective response to reduce glucose uptake by skeletal muscle, thereby favoring its uptake by the brain. However, some regions of the brain, such as the hippocampus, hypothalamus, striatum, and sensorineural cortex use insulin for glucose uptake, which is mediated by insulin receptor-A (IR-A) and involves glucose transporters-4 and 8 (Glut4 and Glut8; Neth and Craft, 2017). When insulin signaling is blocked to these neurons, it signals hunger and food intake. Of interest, chronic fructose ingestion has been found to reduce the activation (phosphorylation) of IR-A and insulin receptor substrate-2 (IRS-2) in the hippocampus of rats (Agrawal et al., 2016). Also, the peripheral insulin resistance is known to raise serum insulin levels which inhibit insulin transporters in the blood-brain barrier and thereby might reduce CNS insulin levels. Some studies also show that peripheral insulin resistance and cerebral glucose hypometabolism frequently coexist, suggesting they are pathogenically linked (Burns et al., 2013). Regardless, in the Alzheimer’s disease patient, there appears to be a decrease in both cerebral insulin levels and the expression of the insulin receptor that occurs early in the course of the disease (Agrawal and Gomez-Pinilla, 2012; Moreira, 2013).

In turn, blocking glucose uptake would be expected to have some negative effects on cerebral energy metabolism (Neth and Craft, 2017) but with the overall benefit to the organism of encouraging the foraging process. Also, the oxidative stress to the mitochondria would further reduce mitochondrial function, and initially, glycolysis would try to compensate, potentially leading to a period of glucose hypermetabolism, as is sometimes observed in early Alzheimer’s disease (Neth and Craft, 2017). However, the neurons need to protect against this oxidative stress, and they do this by limiting the glycolysis and shunting the glucose *via* the pentose phosphate shunt to generate reduced glutathione (Herrero-Mendez et al., 2009). This then leads to cerebral hypometabolism and low intracellular ATP levels.

Consistent with the fructose pathway, early-onset alzheimer’s disease is associated with glycolysis, lactate production, oxidative stress, reduced mitochondrial respiration, ammonia generation, and depressed cerebral glucose metabolism (Hoyer et al., 1988, 1990; Cenini and Voos, 2019). A marker of mitochondrial oxidative stress (and especially from fructose or uric acid), aconitase activity, can be shown to be decreased in circulating lymphocytes of patients with Alzheimer’s disease (Mangialasche et al., 2015). As the disease progresses cerebral oxygen consumption falls (Hoyer et al., 1991) along with a stepwise reduction in cerebral ATP from about 7% in the early stages to more than 50% late in the course (Hoyer, 1992). As mitochondrial density falls, some mitochondria may attempt compensation by accelerating the oxidative phosphorylation (inverse Warburg effect; Demetrius et al., 2014) but over time the progressive lack of cellular energy causes the demise of the neuron.

#### Step 5: Formation of Amyloid Plaques and Neurofibrillary Tangles

A direct mechanism whereby the fructose metabolism pathway can drive the production of amyloid plaques and neurofibrillary tangles is not fully clear. Peripheral insulin resistance is associated with increased cerebral amyloid (Morris et al., 2016), but whether this association carries causality is not known. Sugar (fructose) intake can cause oxidative stress to the pancreatic islets, resulting in hyalinosis and injury of the islets (Roncal-Jimenez et al., 2011), and the latter can increase expression of amylin, a precursor to amyloid proteins (Hayden and Tyagi, 2001). Theoretically, if amylin were released, it could pass through the blood-brain barrier where it might interact with the Aβ amyloid to generate amylin amyloid (Jackson et al., 2013).

One potential mechanism could involve fructose-dependent impairment of insulin signaling (Imamura et al., 2020) with suppression of sirtuin 1 in the hippocampus (Agrawal et al., 2016), which is known to impair the production of heat shock proteins (Westerheide et al., 2009). Heat shock proteins have a role in repairing misfolded proteins, and impaired HSP responses may predispose to the accumulation of tau protein and amyloid in patients with Alzheimer’s disease (Chen et al., 2014; Despres et al., 2017).

Consistent with these ideas, the administration of a high sugar diet in a mouse model of Alzheimer’s disease led to a significant increase in serum and brain amyloid levels (Yeh et al., 2019). More importantly, in a cross-sectional study in cognitively normal older adults, subjects on high glycemic carbohydrates and/or high sugar diets had higher cerebral amyloid deposition as measured by positron emission tomography (PET scan), and those subjects on a high sugar intake also performed worse on the Mini-Mental State Examination (Taylor et al., 2017).

The disturbances in cell energy metabolism pose a heavy toll for the maintenance of neuronal connectivity and function which can accelerate the pathogenesis of neurodegenerative diseases such as Alzheimer’s disease (Gomez-Pinilla and Yang, 2018). Synaptic communication is essential for neuronal function and cognition and highly demanding on energy such that disruptions in cell metabolism associated with fructose can heavily damage overall brain function, beyond simply cell survival. The capacity of the hippocampus to sustain synaptic plasticity in the forms of long-term potentiation (LTP) and long-term depression (LTD)—electrophysiological correlates of learning and memory have shown to be seriously compromised by fructose feeding (Cisternas et al., 2015; Agrawal et al., 2016).

## Limitations

The pathogenesis of Alzheimer’s disease is complex and involves multiple genetic and environmental factors, and our purpose is to present a new hypothesis that links mitochondrial dysfunction, cerebral energetics, cerebral insulin resistance, and diet that might encourage further research. We do not negate the role of other factors, such as varicella-zoster viral infection, that may induce similar pathways (Bubak et al., 2019). We also recognize that the role of dietary fats is complex and that the balance of omega3 to omega6 may also be important, and how this relates to fructose metabolism requires further study (Simopoulos, 2013).

## Summary

We hypothesize that Alzheimer’s disease is driven largely by western culture that has resulted in excessive fructose metabolism in the brain. The fructose metabolism was originally meant to provide a survival benefit by stimulating foraging behavior and reducing energy and oxygen demands. Unfortunately, chronic stimulation in the brain leads to mitochondrial oxidative stress and local inflammation and a progressive reduction in cerebral energy levels. While other tissues increase glycolysis to compensate for the reduced ATP, in neurons glucose is directed to the pentose phosphate shunt to generate antioxidants to combat oxidative stress-induced mitochondrial loss. As a consequence, glucose hypometabolism increased oxidative stress, and a progressive loss of mitochondria occur, leading eventually to neuronal dysfunction and death. In this scenario, the amyloid plaques and neurofibrillary tangles are part of the inflammatory response and participate in injury, but are not the central factors driving the disease. Theoretically, regulating KHK-C in the brain, or regulating AMPD2, might provide novel ways to prevent and treat Alzheimer’s disease.

## Data Availability Statement

The original contributions presented in the study are included in the article, further inquiries can be directed to the corresponding author.

## Author Contributions

RJ wrote the first draft. All authors contributed to the article and approved the submitted version.

## Conflict of Interest

RJ, DT, LS-L, and ML have equity with Colorado Research Partners, LLC, that is developing inhibitors of fructose metabolism for metabolic and alcohol use disorders. RJ also has equity in XORTX Therapeutics that is developing novel xanthine oxidase inhibitors. The remaining authors declare that the research was conducted in the absence of any commercial or financial relationships that could be construed as a potential conflict of interest. The reviewer ZZ declared a past co-authorship with one of the authors FG-P to the handling Editor.
